# The effect of sexual counseling on depression, anxiety, stress, sexual knowledge and sexual quality of life in men who have undergone invasive coronary interventions: An RCT

**DOI:** 10.18502/ijrm.v19i11.9912

**Published:** 2021-12-13

**Authors:** Seyyed Mohsen Sadatinejad, Alireza Farokhian, Mohsen Taghadosi, Seyed Gholamabbas Mosavi

**Affiliations:** ^1^Pediatric Group, Bahrami Children Hospital, Tehran University of Medical Sciences, Tehran, Iran.; ^2^Students Research Committee, Kashan University of Medical Sciences, Kashan, Iran.; ^3^Internal Medicine Group, Medicine Faculty, Kashan University of Medical Sciences, Kashan, Iran.; ^4^Medical Surgical Nursing, Trauma Nursing Research Center, Faculty of Nursing and Midwifery, Kashan University of Medical Sciences, Kashan, Iran.; ^5^Biostatistics Group, Health Faculty, Kashan University of Medical Sciences, Kashan, Iran.

**Keywords:** Percutaneous transluminal coronary angioplasty, Coronary artery bypass graft, Life quality, Depression, Anxiety neuroses, Sex counseling.

## Abstract

**Background:**

Sexual dysfunctions are common in men with ischemic heart disease, especially in men undergoing therapeutic interventions.

**Objective:**

This study intended to assess the effect of counseling on depression, anxiety, stress, sexual knowledge and sexual quality of life in men after invasive coronary interventions in the post catheterization department of Kashan Shahid Beheshti Hospital during 2018.

**Materials and Methods:**

The study population consisted of 54 male participants who had undergone an invasive coronary intervention. The intervention group received counseling and the control group underwent the standard ward routine. Data were collected using the depression anxiety stress scales, Abraham's sexual quality of life, and the Yi-Hung Sexual Knowledge questionnaires before discharge and also two months later.

**Results:**

Within two months, the intervention group's mean score of sexual knowledge significantly increased, compared with the control group, from 12.37 to 14.81 (p ≤ 0.001). The intervention group's mean score of sexual quality of life also significantly increased, compared with the control group, from 48.2 to 60.7 (p ≤ 0.001). Moreover, the mean anxiety score changed in the intervention group from 11.18 to 5.25, again a significant difference compared with the control group (p = 0.01). But, the differences in the depression and stress scores were not significant.

**Conclusion:**

Our findings suggest that sexual counseling may improve sexual knowledge, sexual quality of life and anxiety in men following invasive coronary intervention, but might not reduce their stress or depression. Further studies are needed to confirm these findings.

## 1. Introduction

Ischemic heart events occur more frequently in men than in women, and occur at younger ages and cause more complications. Sexual dysfunction is a common complication of cardiac events (experienced by over 93% of patients) which may lead to imbalances in the interpersonal relations and affect their quality of life (1, 2). Also, their medications may cause sexual dysfunction (3). Sexual dysfunction occurs in more than half of individuals undergoing open-heart surgery (4). Studies have indicated that 47–85% of patients, mostly men, experience sexual dysfunction after invasive coronary interventions (5-9), and in some cases these complications increase with angioplasty or coronary artery bypass graft (CABG) (10, 11).

Treatment of mental health issues may affect the sexual dysfunction (12). In this regard, education and improvement of couples' relationships are among the therapeutic methods used. Additionally, providing education in the hospital is a key point (13). Some of the mental health issues (such as stress and anxiety) are caused by a lack of knowledge and information on appropriate sexual and intimate relationships and a lack of physician recommendations before discharge (14).

Studies have reported varied results on sexual counseling. Overemphasis on the increased risk of cardiac attack during coitus by the health care team in sexual counseling may cause sexual dysfunction (15). On the other hand, excessive reassurance may bring harmful consequences by leading to cardiac attacks and readmissions, which will highly affect future sexual relationships. Some counseling interventions improve sexual knowledge, performance, and satisfaction and early patient recovery to normal sexual activities after cardiac events (12) while some results demonstrate the opposite effect (15, 16).

Given the potential positive effects of sexual dysfunction treatment on improving the sexual quality of life, the early diagnosis and treatment of these complications is important (17).

Due to the low sexual quality of life of many cardiac patients (16), increased use of invasive interventions for cardiovascular diseases (10), widespread ignorance about patients' sexual disorders (18), lack of knowledge and communication skills of health care staff surrounding sexual counseling (14), common concerns of men with cardiovascular disease about their sexual life (19), and lack of comprehensive guidance for sexual counseling of patients with cardiovascular diseases (20), this study was designed to assess the effect of sexual counseling before discharge on sexual knowledge, sexual quality of life, stress, anxiety, and depression in men that have undergone invasive coronary interventions. The results of this study could be used in the rehabilitation of men with cardiovascular diseases to improve their sexual quality of life.

## 2. Materials and Methods

This was a randomized controlled trial which involved 156 male participants who had undergone invasive coronary interventions (CABG or percutaneous transluminal coronary angioplasty) in the post catheterization department of Shahid Beheshti Hospital (Kashan, Iran) from April to September 2018. A continuous sampling method was used. Participants were allocated to the intervention or control group randomly by random computer numbers (performed by the researcher using a calculator). As is convention, multiples of the number two placed the participants in the intervention group, and other numbers placed the participants in the control group.

The inclusion criteria were: married men (living with their wife), aged 35-65 yr, who had undergone invasive coronary interventions. The exclusion criteria were: heart, liver, pulmonary or kidney failure; malignancy; any systemic disease; use of antipsychotic or cytotoxic drugs; severe heart failure (function class III or IV (by New York Heart Association classification)); alcohol or opium abuse (reported by the participants); current depression, anxiety, or stress (based on a DASS-21 score of < 10 for depression, < 8 for anxiety or < 15 for stress); current known sexual dysfunction; or current known psychological disorder.

**Figure 1:**
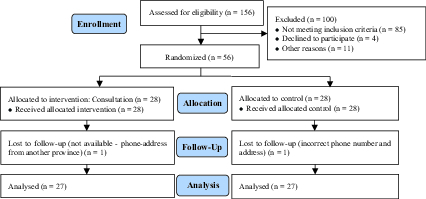
Consort chart.

Details are shown in the consort diagram (Figure 1). Demographic information such as age, sex, level of education, history of cardiovascular interventions, occupation, underlying diseases, and medications were recorded, with a focus on confidentiality. Self-reported questionnaires were given to the participants. The questionnaires used were the DASS-21 depression, anxiety, and stress scale, Abraham's Sexual Quality of Life scale (21), and a sexual knowledge scale (4).

Abraham's Sexual Quality of Life Scale (for men) has been validated in Persian with a content validity index of 0.87 and a Cronbach's alpha of 0.82 (21). This scale consists of 11 items, and each item can be rated 1 to 6 points (Likert scale); higher points indicate a higher quality of life. The sexual knowledge scale that was used is a 20-item scale for men after invasive coronary interventions; its content validity and reliability were shown by Lai et al. (4). Participants were asked to answer questions about sexual knowledge as true or false. The correct answer scored a 1, whereas an incorrect answer was scored as 0. Total scores ranged from 0 to 20, with higher scores indicating greater sexual knowledge. This sexual knowledge scale (4) had a content validity index of 0.78 and a Cronbach's alpha of 0.73. During translation into the Persian language, three questions (items 18-20) were eliminated as part of cultural adaptation. The reliability of the tool was rechecked and the new Cronbach's alpha was 0.72 (22). The DASS-21 score validity and reliability have already been demonstrated (23).

The questionnaires were completed twice: before discharge and again two months after discharge (to record prolonged changes). For illiterate participants, the researchers wrote down their exact words.

After completing the first questionnaire, participants in the intervention group received sexual counseling along with routine advice, while participants in the control group underwent routine discharge procedures (at a similar meeting, they received the usual health advice). In the intervention group, patients were counseled for 20 min by the researcher and then they were given a booklet and DVD with additional information adapted from authoritative cardiovascular references (24-27) approved by cardiologists and cardiac nurses (Table I). We tried not to let the groups know about each other's training program. The face-to-face counseling session discussed the information in the booklet and participants' questions were clearly answered. The counseling took place before discharge to reduce bias due to participants' interactions.

After discharge, the researcher communicated with the participants several times through telephone calls and virtual networks (e.g. WhatsApp) to ensure the participants used the booklet and DVD. Also, participants could ask the researcher any questions. After two months, the same questionnaires were completed again (by telephone call or in-person session).

**Table 1:** The topics discussed in the booklet and DVD


	**Topics**
**1**	Indications and duration for abstinence from sexual intercourse for participants after different procedures
**2**	Difference between normal and abnormal heart and respiratory rate and chest pains during sexual activity in men with cardiac disease
**3**	Medications that reduce or improve sexual function
**4**	Recommendations for lifestyle like physical exam, number and content of meals, and not smoking
**5**	Recommendations for appropriate sexual activity (time, duration, positions, alarm signs)
**6**	When to contact a physician or go to the emergency ward
**7**	Strategies for the relief of stress and anxiety before sexual activity

### Ethical considerations

The study was approved by the Kashan University of Medical Science Research Ethics Committee (Code: IR.KAUMS.MEDNT.REC.1396.97). Informed consent was taken before the intervention and confidentiality was prioritized. Participants were consulted alone to maintain confidentiality and collected data were kept anonymous. Participants could withdraw from the study at any time. In accordance with participants' rights, participants in the control group received a 20- to 30-minute sexual counseling session by telephone after filling out the questionnaires for the second time.

### Statistical analysis

The data were analyzed using the Statistical Package for the Social Sciences (SPSS, version 16, IBM Company), by Chi-square, *t* test, paired *t* test, Leven test, Fisher's exact test, Kolmogorov-Simonov, Wilcoxon, and ANCOVA. A significance level of p < 0.05 was used.

## 3. Results

Of the 156 participants initially deemed eligible, 85 did not meet the inclusion criteria. Also 11 participants were excluded due to mental health conditions or sexual dysfunction and four participants did not consent to participate in the study. Two participants could not be contacted for the two-month follow-up (one in the intervention group and one in the control group). Finally, 54 participants remained in the study.

Participants of the control and intervention groups were not significantly different in terms of the level of education, occupation, residence, smoking status, history of invasive interventions, underlying diseases (such as diabetes mellitus, hypertensions and hyperlipidemia), or drug classes of previous prescribed medications (Table II).

Covariance analysis was performed on the sexual knowledge score data. As demonstrated in table III, this analysis showed that sexual counseling seemed to affect the sexual knowledge scores (f = 11.13, p ≤ 0.01). The difference in sexual quality of life scores before and two months after counseling was measured and compared (Table III). This analysis showed that the sexual quality of life score was significantly related to the counseling (t = 2.98, p ≤ 0.01).

The depression scores two months after discharge were not normally distributed. The difference in depression scores before and two months after counseling was measured and compared (Table IV), which showed that depression was not affected by sexual counseling. Covariance analysis was also performed for the anxiety and stress data (Table IV). This analysis showed that sexual counseling affected anxiety scores (f = 7.018, p = 0.01) but there was no relationship between counseling and stress scores.

**Table 2:** Demographic information of the participants


** Variable**		**Control group **	**Intervention group **	**p-value**
** Job**				
	**Freelance**	7 (25.9)	8 (29.6)	
	**Employee**	1 (3.7)	1 (3.7)	
	**Farmer**	4 (14.8)	3 (11.1)	
	**Others**	15 (55.6)	15 (55.6)	1.00 ${}^{a}$
** Income in million tomans**				
	** < 1.5**	19 (70.4)	22 (81.5)		
	**1.5-2.5**	6 (22.2)	4 (14.8)		
	** > 2.5**	2 (7.4)	1 (3.7)	0.62 ${}^{a}$
** Level of education**				
	**Elementary**	3 (11.1)	3 (11.1)		
	**Intermediate (pre-diploma)**	15 (55.6)	17 (63.0)	
	**Diploma and above**	9 (33.3)	7 (25.9)	0.97 ${}^{a}$
** Residence**				
	**Urban**	24 (88.9)	25 (92.6)		
	**Rural**	3 (11.1)	2 (7.4)	≈ 1.00 ${}^{a}$
** Smoking**		21 (77.8)	24 (88.9)		0.47 ${}^{a}$
** Underlying disease**				
	**Diabetes**	10 (37.0)	14 (51.9)	0.27 ${}^{b}$
	**Hyperlipidemia**	17 (63.0)	15 (55.6)	0.58 ${}^{b}$
	**Hypertension**	16 (59.3)	18 (66.7)	0.57 ${}^{b}$
** History of cardiovascular intervention**		11 (40.7)	13 (48.1)	0.58 ${}^{b}$
** Medications**				
	**Nitrates**	10 (37.0)	9 (33.3)	0.78 ${}^{b}$
	**Beta-Blockers**	9 (33.3)	9 (33.3)	1.00 ${}^{b}$
	**ARBs**	12 (44.4)	13 (48.1)	0.79 ${}^{b}$
	**ACEIs**	2 (7.4)	2 (7.4)	1.00 ${}^{a}$
	**Astatines**	13 (48.1)	17 (63.0)	0.27 ${}^{b}$
	**Thiazides**	0 (0)	1 (3.7)	1.00 ${}^{a}$
Data presented as n (%). ${}^{a}$ Fisher's exact test, ${}^{b}$ Chi-square test. ARB: Angiotensin receptor blockers, ACEI: Angiotensin converting enzyme inhibitors				

**Table 3:** Sexual knowledge and sexual quality of life scores before discharge and two months later


** Study group**		**Before discharge**	**Two months after discharge**	**p-value**	**p-value (between groups)**
** Sexual knowledge score**					
	**Intervention**	12.37 ± 2.38	14.81 ± 1.03	≤ 0.01*	
	**Control**	11.62 ± 2.37	13.62 ± 1.41	≤ 0.01*	≤ 0.01***
	** Sexual quality of life score**					
	**Intervention**	48.20 ± 12.03	60.70 ± 10.39	≤ 0.01**	
	**Control**	49.44 ± 17.25	51.48 ± 14.68	0.37*	≤ 0.01*
	Data presented as Mean ± SD. *Paired *t* test, **Wilcoxon test, ***ANCOVA					

**Table 4:** Depression, anxiety, and stress scores before discharge and two months later


** Study group**		**Before discharge**	**IQR**	**Two months after discharge**	**IQR**	**p-value**	**p-value (between groups)**
** Depression score**							
	**Intervention**	14.07 ± 11.61	11	3.92 ± 6.23	4	≤ 0.01**	
	**Control**	16.66 ± 12.94	11	8.29 ± 10.52	7	≤ 0.01*	0.62*
	** Anxiety score**							
	**Intervention**	11.18 ± 8.17	5	5.25 ± 4.64	3	≤ 0.01*	
	**Control**	9.18 ± 10.30	8	9.85 ± 8.76	8	0.76*	0.01***
	** Stress score**							
	**Intervention**	17.70 ± 10.83	10	7.18 ± 7.75	6	≤ 0.01*	
	**Control**	21.55 ± 12.53	12	13.11 ± 12.56	11	≤ 0.01*	0.67***
	Data presented as Mean ± SD. *Paired *t* test, **Wilcoxon test, ***ANCOVA, IQR: Interquartile range							

## 4. Discussion

In the present study, the mean sexual knowledge score in the intervention group increased from 12.37 to 14.81 (Table III). A systematic review reported that in some cases, counseling improved sexual knowledge, performance, and satisfaction while in the other case, counseling did not make a difference (28). Several studies have shown that the sexual education of patients with cardiovascular diseases improved their sexual performance (12, 13, 29). Another study reported that most patients leave the hospital without their questions on sexual relationships being answered. Most patients in their study were willing to receive information on sexual activity after discharge, while 69% of them did not receive any sexual counseling before discharge (18).

In the present study, the mean sexual quality of life score in the intervention group increased from 48.2 to 60.7 (Table III). Several studies have shown that invasive cardiac interventions improved the quality of life score of patients with cardiovascular diseases within several months, though this increase was not significant (30) or the effect on the sexual quality of life was unclear in their study (31). We can infer that sexual satisfaction improvement required sexual counseling and rehabilitation in one study (9).

Studies have reported up to 85% sexual dysfunction in patients undergoing invasive interventions (6). Sexual knowledge is an important factor regarding the sexual quality of life after invasive cardiac interventions, i.e. some studies stated that poor sexual knowledge can highly affect sexual health, performance, satisfaction, and quality (4, 32). Inappropriate sexual knowledge and activity is one of the main causes of readmission within six months after discharge, which is partly caused by a lack of patient awareness of the appropriate approach to coitus after discharge (33).

Some researchers have suggested initiating sexual counseling as soon as possible in the hospital before patient discharge (13), as was done in this study.

The results of this study indicated that mean anxiety scores in the intervention group decreased from 11.18 to 5.25 but while this change was significant, no significant difference was observed regarding the stress and depression scores (Table IV).

Fear was the main cause of reduced sexual satisfaction and improved knowledge significantly reduced fear (34). Sexual education and rehabilitation of men with cardiovascular diseases may reduce stress, anxiety, and depression (35). But, there are some controversies that sexual counseling can reduce the sexual satisfaction due to increased fear and stress, if the counseling overemphasizes the high risk of cardiac attack during coitus (15). These differences in findings may be attributed to the different content and presentation of the counseling sessions.

Studies have demonstrated that organic factors such as erectile dysfunction only account for 15-20% of sexual dysfunction and the other cases are related to psychological factors such as stress and anxiety (5). Psychological factors may be resolved by increasing sexual knowledge, though for many reasons sexual issues are ignored in hospitals by the health care team (14). Psychological issues such as fear can significantly affect the sexual quality of life. Wives of many patients with cardiovascular disease are scared of the consequences of sexual activity which may lead to avoidance of sexual relationships and anger towards a patient's health (36). Failure to reduce stress and depression may be due to counseling that is performed by an unreliable, inexperienced counsellor and social taboos of sexual issues; future studies are needed to investigate this. The reduced stress scores demonstrated in both groups (Table IV) may be related to the passage of time after a stressful procedure (percutaneous transluminal coronary angioplasty/CABG). We suggest that further studies evaluate stress over multiple short time periods of follow-up after consult (due to the rapid variability in stress with daily events and short-term stress responses to consultations). Also, we suggest longer follow-up studies are conducted with several sessions of counseling for evaluating depression.

## 5. Conclusion

Face-to-face sexual counseling combined with an educational video and booklet increased sexual knowledge and sexual quality of life and reduced patient anxiety scores in the intervention group compared with the control group, but stress and depression scores were not significantly changed. Thus, we recommend that cardiologists acquire sexual counseling skills to perform sexual counseling along with the standard medical treatment in the rehabilitation of patients. Further studies are needed to investigate the impact of counseling on stress and depression.

##  Conflict of Interest

The authors declare that they have no conflict of interest.
